# Rapid assessment of the effect of ciprofloxacin on chromosomal DNA from *Escherichia coli *using an in situ DNA fragmentation assay

**DOI:** 10.1186/1471-2180-9-69

**Published:** 2009-04-13

**Authors:** María Tamayo, Rebeca Santiso, Jaime Gosalvez, Germán Bou, José Luis Fernández

**Affiliations:** 1 INIBIC-Complejo Hospitalario Universitario A Coruña, Unidad de Genética, As Xubias 84, 15006- A Coruña, Spain; 2 Laboratorio de Genética Molecular y Radiobiología, Centro Oncológico de Galicia, Avda. de Montserrat s/n, 15009-A Coruña, Spain; 3 Unidad de Genética, Facultad de Biología, Universidad Autónoma de Madrid, 28049-Madrid, Spain; 4 INIBIC-Complejo Hospitalario Universitario A Coruña, Servicio de Microbiología, As Xubias, 84, 15006-A Coruña, Spain

## Abstract

**Background:**

Fluoroquinolones are extensively used antibiotics that induce DNA double-strand breaks (DSBs) by trapping DNA gyrase and topoisomerase IV on DNA. This effect is usually evaluated using biochemical or molecular procedures, but these are not effective at the single-cell level. We assessed ciprofloxacin (CIP)-induced chromosomal DNA breakage in single-cell *Escherichia coli *by direct visualization of the DNA fragments that diffused from the nucleoid obtained after bacterial lysis in an agarose microgel on a slide.

**Results:**

Exposing the *E. coli *strain TG1 to CIP starting at a minimum inhibitory concentration (MIC) of 0.012 μg/ml and at increasing doses for 40 min increased the DNA fragmentation progressively. DNA damage started to be detectable at the MIC dose. At a dose of 1 μg/ml of CIP, DNA damage was visualized clearly immediately after processing, and the DNA fragmentation increased progressively with the antibiotic incubation time. The level of DNA damage was much higher when the bacteria were taken from liquid LB broth than from solid LB agar. CIP treatment produced a progressively slower rate of DNA damage in bacteria in the stationary phase than in the exponentially growing phase. Removing the antibiotic after the 40 min incubation resulted in progressive DSB repair activity with time. The magnitude of DNA repair was inversely related to CIP dose and was noticeable after incubation with CIP at 0.1 μg/ml but scarce after 10 μg/ml. The repair activity was not strictly related to viability. Four *E. coli *strains with identified mechanisms of reduced sensitivity to CIP were assessed using this procedure and produced DNA fragmentation levels that were inversely related to MIC dose, except those with very high MIC dose.

**Conclusion:**

This procedure for determining DNA fragmentation is a simple and rapid test for studying and evaluating the effect of quinolones.

## Background

Fluoroquinolones are broad-spectrum antibacterial agents that are used widely to treat a variety of infections, such as gonococcal infections, osteomyelitis, enteric, and respiratory and urinary tract infections. Ciprofloxacin (CIP) is one of the most consumed fluoroquinolones worldwide [1,2]. The type II topoisomerases DNA gyrase and topoisomerase IV are the target of quinolones [3,4]. DNA gyrase is the preferential target in gram-negative bacteria such as *E. coli*, whereas topoisomerase IV is affected mainly in gram-positive bacteria [5]. These enzymes induce transient DNA double-strand breaks (DSBs) on bacterial chromosomes, which either introduce negative supercoiling, as in the case of DNA gyrase, or relax supercoiling and decatenate-replicated daughter chromosomes, as in the case of topoisomerase IV [3-5]. DNA gyrase is a tetramer with two GyrA and two GyrB subunits, and topoisomerase IV comprises two ParC and two ParE subunits. After DSB induction, the topoisomerase passes through the DNA duplex, seals the break, and releases DNA. During this process, a transient covalent link is established between the GyrA or the ParC subunits and the 5' end of each DNA break [3,5].

Quinolones bind rapidly to the DNA topoisomerases attached to DNA, producing ternary complexes comprising quinolone-topoisomerase-DNA. These complexes promptly block DNA replication and RNA transcription, an action that inhibits cell growth but does not clearly explain the cell killing by quinolones [5-7]. After formation of the ternary complex, a DSB is produced by topoisomerase, but bound quinolone inhibits the subsequent ligation of the DNA ends by trapping the topoisomerase on DNA to the GyrA or ParC proteins through a covalent bond of the 5' ends. These products are called cleaved complexes and are distributed throughout the bacterial chromosome. When using first-generation quinolones such us nalidixic acid, DSBs are constrained initially by the proteins from the cleaved complexes, and this process can be reversed by removing the quinolone, adding EDTA, or mild heat treatment. Cell killing is relatively slow, and the rate of killing seems to correlate with later massive chromosomal DNA fragmentation mediated by a putative protein suicide factor, whose synthesis may be blocked by chloramphenicol. In contrast, a high concentration of fluoroquinolones such as CIP or gatifloxacin produces rapid cell death and chromosomal DNA fragmentation, processes that are not protected by chloramphenicol and thus are protein synthesis independent [6,7]. In this case, DSBs from the cleaved complexes behave as irreversible products possibly because of the drug-mediated dissociation of topoisomerase subunits, and the DNA breaks are released from the protein constraint, thereby fragmenting the chromosome. Bactericidal antibiotics, including the quinolone norfloxacin, may induce the production of hydroxyl radicals that can cause extensive oxidative cellular damage, including secondary DNA injury, which may contribute to bacterial death [8,9].

Quinolone resistance results essentially from target modification caused by mutations in the genes encoding the subunits of DNA gyrase and topoisomerase IV, especially in the quinolone resistance-determining region (QRDR) [10-12]. Several mutations may coexist in the same or in different subunits and may produce high-level resistance. Changes in drug permeation or overexpression of efflux pumps may also be involved and, in combination with QRDR mutations, may contribute to high-level resistance [10-12]. Several recent studies indicate that target protection through plasmid-mediated quinolone-resistance genes also may play a significant role, and its prevalence is increasing worldwide [13]. The existence of fluoroquinolone-inactivating enzymes, like a variant of the gene that encodes aminoglycoside acetyltransferase AAC(6')-Ib, has been proposed [14]. This enzyme variant would reduce the activity of both aminoglycosides and CIP.

Given the extended use of fluoroquinolones, especially CIP, a more thorough understanding of their activity is needed. Because chromosomal DNA fragmentation is the main mechanism that correlates with cell killing [5-7], it is the parameter of choice to assess fluoroquinolone activity. We have recently developed a kit that allows the simple and rapid assessment of the presence of fragmented DNA at the single-cell level in micro-organisms [15]. Cells immersed in an inert microgel on a slide are lysed, stained with a highly sensitive DNA fluorochrome, and visualized with a fluorescence microscope. The nucleoids with fragmented DNA are discriminated clearly by their peripheral halo of diffused DNA fragments. The greater the fragmentation, the greater the number of DNA spots and the greater the circular surface area of diffusion evident in this assay. Here we show the significant technical value of our procedure for determining the activity of fluoroquinolones, particularly for detecting chromosomal DNA damage and repair after CIP treatment in *E. coli*.

## Methods

### Cultures

Chromosomal DNA fragmentation in situ was assayed in the TG1 *E. coli *strain, which was grown routinely in Luria Bertani (LB) broth (1% Bacto-tryptone, 0.5% yeast extract, 0.5% NaCl) or on LB agar at 37°C in aerobic conditions. *E. coli *TG1 [genotype: F *traD36 LacI*q (*lacZ*)*M15*] *proAB*/*supE *(*hsdMmcrB*)5(*r*k*m*k *McrB*) *thi *(*lac*-*proAB*). Cell growth in liquid cultures was evaluated by monitoring turbidity at OD_600 _using a spectrophotometer (Unicam 8625, Cambridge, UK). The minimum inhibitory concentration (MIC) was determined using the E-test (AB Biodisk, Solna Sweden) according to manufacturer's instructions. Viability was determined by colony counting after sequential dilutions and plating. To determine the percentage of viable cells, the number of cells seeded on the plate was counted using a cytometric camera.

### Experiments

Three different experiments were performed with TG1 *E. coli*, all in triplicate. Typical experiments are presented. In the first, several colonies of TG1 *E. coli *were grown overnight on LB agar plates and then resuspended in LB broth at an OD_600 _of 0.05 and grown to an OD_600 _of 0.8. The colonies were then incubated with 0, 0.003, 0.006, 0.008, 0.012, 0.02, 0.04, 0.08, 0.1, 0.5, or 1 μg/ml CIP (Sigma) in 15 ml Falcon tubes containing 4 ml of LB broth for 40 min at 37°C with aeration and shaking, and then processed to measure the chromosomal DNA fragmentation.

In the second experiment, TG1 *E. coli *was removed from culture in LB agar, resuspended in LB broth at an OD_600 _of 0.5, and treated with 1 μg/ml CIP in LB broth at 37°C with aeration and shaking. Aliquots were removed after 0, 5, 10, 15, 20, 30, and 40 min of incubation, and processed to measure DNA fragmentation. The time needed to prepare the microgel with the cells enclosed, before the slide was immersed in the lysing solution, was 8 min (see next section). In the results, this time must be added to each incubation period. To complete this experiment, TG1 *E. coli *were cultured in liquid LB broth at 37°C for 23 h with aeration and shaking, and the growth was monitored by measuring the turbidity (OD_600_). The liquid cultures started at an OD_600 _of 0.05. Aliquots were removed during the exponentially growing phase at 3 h (i.e., at an OD_600 _of 0.52) and during the stationary phase at 7 h (OD_600_: 1.20), 9 h (OD_600_: 1.52) and 23 h (OD_600_: 1.84). At the end of each designated time, 1 μg/ml of CIP was added directly to the aliquot, and the aliquot was incubated at 37°C for 0 and 5 min, and then processed to measure the DNA fragmentation.

In the third experiment, the micro-organisms were grown overnight on LB agar plates, resuspended in LB broth at an OD_600 _of 0.05, grown to an OD_600 _of 0.8, and then incubated with 10, 1, or 0.1 μg/ml CIP in LB broth for 40 min at 37°C. After the incubation, the CIP was removed from the medium by centrifuging the bacteria and washing in plain LB broth. The bacteria were incubated at 37°C in LB broth with aeration and shaking, and aliquots were removed at 0, 1.5, 3, 4, 5, and 24 h. For the 0.1 μg/ml dose of CIP, the bacteria were also incubated for 6 h. One aliquot was used to measure the DNA fragmentation, and another was plated on LB agar at 37°C to measure the viability after 24 h of culture. Cultures without CIP and with CIP incorporated in the new LB medium added after washing after the initial CIP treatment were included and processed along with each dose and for the various incubation times.

### Bacterial strains with low CIP sensitivity

Besides the experiments with TG1, DNA fragmentation was measured in four *E. coli *strains whose low sensitivity to CIP and underlying mechanisms are known. These included strains with mutations in the QRDR region from GyrA and ParC [16]. The isolates were C-15 (Ser83Leu from GyrA; CIP MIC = 0.25 μg/ml); 1273 (Ser83Leu and Asp87Tyr from GyrA; CIP MIC: 8.0 μg/ml), and 1383 (Ser83Leu and Asp87Tyr from GyrA together with Ser80Ile and Glu84Lys from ParC; CIP MIC: 128 μg/ml), and the control strain C-20 with no mutation in the QRDR region (CIP MIC: 0.007 μg/ml). The strain J53 with the plasmid-mediated quinolone-resistance gene qnrA1 (CIP MIC: 0.25 μg/ml) and its control strain J53 without the plasmid were also examined [17]. These strains were exposed to CIP at the MIC dose, at 10× and 100× the MIC dose, and at 0.5× and 0.25× the MIC dose for 40 min at 37°C in the exponentially growing phase, and DNA fragmentation was determined.

### Determination of DNA fragmentation

The Micro-Halomax^® ^kit for fluorescence microscopy (Halotech DNA SL, Madrid, Spain) was used. A thorough description has been published previously [15]. Essentially, an aliquot of each sample was diluted to a concentration of 5–10 million micro-organisms/ml in LB medium. The kit includes 0.5 ml snap cap microfuge tubes containing gelled aliquots of low-melting point agarose. The tube was placed in a water bath at 90–100°C for about 5 min to melt the agarose completely and then placed in a water bath at 37°C. Twenty-five microlitres of the diluted sample was added to the tube and mixed with the melted agarose. A 20 μl aliquot of the sample-agarose mixture was pipetted onto a precoated slide, and the sample was covered with a 22 mm × 22 mm coverslip. The slide was placed on a cold plate in the refrigerator (4°C) for 5 min to allow the agarose to produce a microgel with the trapped intact cells inside. The coverslip was removed gently, and the slide was immediately immersed horizontally in 10 ml of the lysing solution for 5 min at 37°C. The slide was washed horizontally in a tray with abundant distilled water for 3 min, dehydrated by incubating horizontally in cold (-20°C) ethanol of increasing concentration (70%, 90%, and 100%) for 3 min each, and air-dried in an oven.

The dried slide was incubated in a microwave oven at 750 W for 4 min, and the DNA was stained with 25 μl of the fluorochrome SYBR Gold (Molecular Probes, Eugene, OR, USA) diluted 1:100 in TBE buffer (0.09 M Tris-borate, 0.002M EDTA, pH 7.5) for 5 min in the dark.

Images were viewed under an epifluorescence microscope (Nikon E800), with a 100× objective and appropriate fluorescence filters, and the images were acquired using a high-sensitivity CCD camera (KX32ME, Apogee Instruments, Roseville, CA, USA). Groups of 16-bit digital images were obtained at each experimental time under similar conditions and stored as TIFF files. Image analysis was performed using a macro designed with Visilog 5.1 software (Noesis, Gif sur Yvette, France). This macro allows for thresholding and background subtraction, and delineates the circular area of diffusion of the DNA fragments from nucleoids. The width delimitated between the edge of the nucleoid and the circumference that limits the circular peripheral area of spreading of DNA fragments is the simplest parameter to estimate DNA fragmentation level after CIP treatment and was measured in μm. At each experimental time, 50–125 nucleoids were evaluated.

### Statistical analysis

Because the data did not follow a normal distribution as ascertained by the Kolmogorov-Smirnov test, the non-parametric Mann-Whitney *U *test was performed to compare the groups. Significance was defined as *P *< 0.05.

## Results

### Dose response

**Figure 1:**
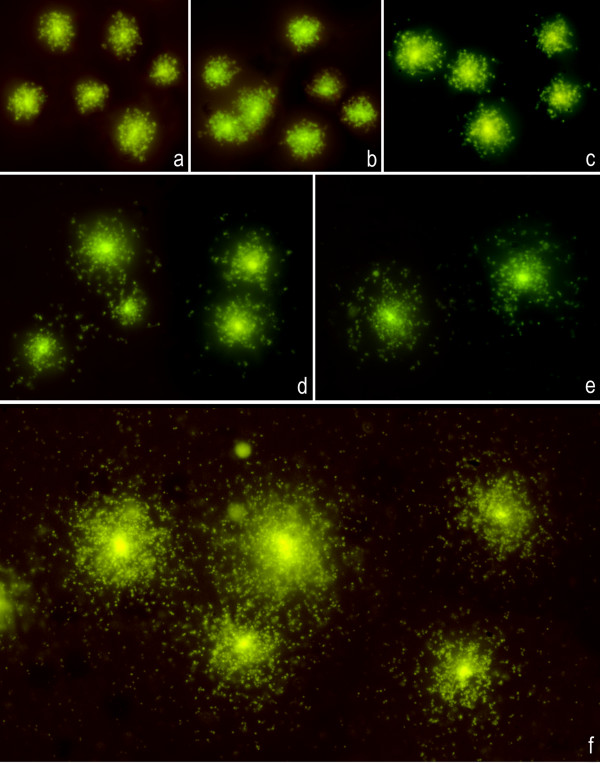
**Dose-response effect of CIP on nucleoids from the *E. coli *strain TG1**. After 40 min incubation with CIP, the cells were processed with the Micro-Halomax^® ^kit and stained with SYBR Gold. **a: **Control untreated cells;**b: **0.008 μg/ml; **c: **0.012 μg/ml, i.e., the MIC dose; **d: **0.04 μg/ml; **e: **0.1 μg/ml; **f: **0.5 μg/ml.

The *E. coli *strain TG1 (CIP MIC of 0.012 μg/ml) was exposed to increasing doses of CIP in liquid LB medium for 40 min at 37°C (Fig. 1). Doses less than the MIC did not result is visible DNA fragments, even after increasing the incubation time with the antibiotic to 90 min (Fig. 1b). The MIC dose resulted in a clear effect: nucleoids appeared compact but with few peripheral DNA fragments (Fig. 1c). As the dose increased, the number of DSBs increased gradually, which was reflected in progressively more DNA fragments and their elevated surface showing peripheral diffusion from the nucleoid (Figs 1d and 1e). After the 0.5 μg/ml dose, all nucleoids appeared massively fragmented as small DNA spots that diffused widely from their original place in the bacteria (Fig. 1f). The 1 μg/ml dose resulted in nucleoids that appeared similar to those obtained after 0.5 μg/ml. The degree of fragmentation tended to be homogeneous after each dose, probably because of the relative similarity in the response to the antibiotic between the different bacteria. The DNA fragments always appeared as small spots, independent of the dose.

**Figure 2:**
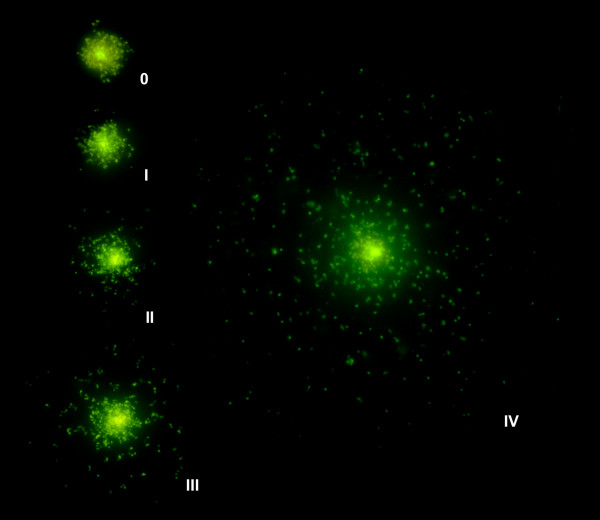
**Nucleoids from *E. coli *strain TG1 with progressively increased DNA fragmentation after incubation with increasing doses of CIP**. 0: undamaged; I: low damage level; II: intermediate damage; III: high damage level; IV: massive fragmentation.

The width of the dispersion of the fragments from the boundary of the nucleoid was quantified using an image analysis system; this measure is a simple and reliable quantitative parameter that reflects the level of CIP-induced DNA damage (Table 1). Differences were significant between the doses tested from 0.012 μg/ml, except between 0.012 μg/ml and 0.02 μg/ml, between 0.04 μg/ml and 0.08 μg/ml, and between 0.5 μg/ml and 1 μg/ml. Using the images obtained, the nucleoids were categorized into five classes of damage, as shown in Fig. 2 and Table 1: class 0: undamaged, dose of 0 to 0.008 μg/ml (Figs 1a and 1b); class I: low damage level, dose of 0.012 or 0.02 μg/ml (Fig. 1c); class II: intermediate level, dose of 0.04 or 0.08 μg/ml (Fig. 1d); class III: high level, dose of 0.1 μg/ml (Fig. 1e); and class IV: massive fragmentation, doses of 0.5 or 1 μg/ml or higher (Fig. 1f). This latter class of damage was practically undistinguishable from that shown by nucleoids with extensive DNA fragmentation always present spontaneously in cultures [15]. Classification into classes is standard practice in mutagenesis studies and provides a perceptive description that is especially useful when heterogeneity in the DNA damage rank is evident between the different nucleoids, as observed in the DNA repair experiments.

**Table 1:** Dose-response effect of CIP on TG1 *E. coli *chromosomal DNA analyzed with the Micro-Halomax^® ^kit.

Dose (μg/ml)	Width of dispersion (μm)	Class	Range
0	-		
		
0.003	-	0	0
		
0.006	-		
		
0.008	-		

0.012	1.3 ± 0.3	I	≤ 2.0
		
0.02	1.6 ± 0.3		

0.04	2.5 ± 0.4	II	2.1 – 3.7
		
0.08	3.3 ± 0.4		

0.1	5.1 ± 1.0	III	3.8 – 5.7

0.5	7.8 ± 1.4	IV	≥ 5.8
		
1	8.8 ± 1.6		

The width of the halo of dispersion of DNA fragments is presented in μm (mean ± standard deviation). The extent of DNA damage was classified according to the width of the dispersion.

### Incubation time

**Figure 3:**
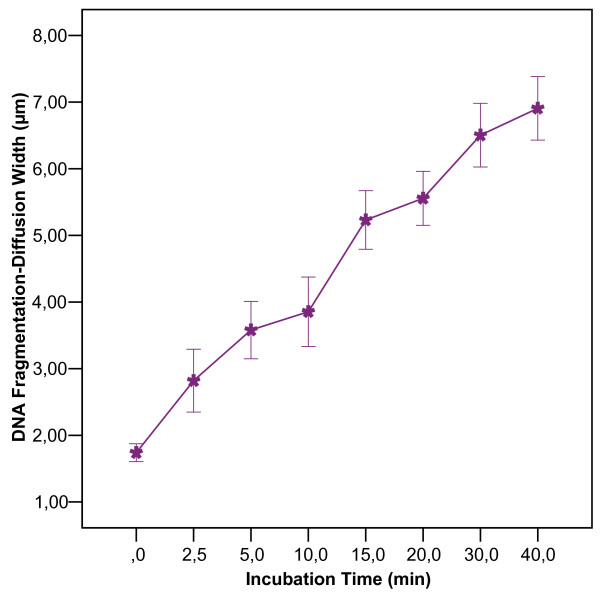
**Effect of the incubation time at a dose of 1 μg/ml of CIP**. The DNA fragmentation level is categorized by the width of the halo of diffusion of the DNA fragments emerging from nucleoids of *E. coli *strain TG1.

To determine the minimum incubation time needed to detect a DNA-breakage effect, the TG1 *E. coli *were collected from LB agar and exposed in liquid LB to 1 μg/ml CIP for 0, 5, 10, 15, 20, 30, and 40 min. The microgel preparation time before immersion in the lysing solution (8 min) must be added to these times because the antibiotic may enter the bacteria and act during this period. Detectable but subtle damage was apparent after 0 min (class I: diffusion width 1.7 ± 0.2 μm) (Fig. 3); this subtle damage appeared as nucleoids with some peripheral DNA fragments unlike in the untreated control cells. As the incubation time increased, the level of DNA damage increased progressively to class II after 2.5 min (2.8 ± 1.0 μm) and class III after 15 min (5.2 ± 1.0 μm); nucleoids appeared massively fragmented after 30 min (class IV, 6.5 ± 1.1 μm) (Fig. 3). As in the dose-response study, the DNA damage intensity also tended to be homogeneous in the different nucleoids at each sample time.

The DNA fragmentation level did not differ between bacteria incubated with the antibiotic at room temperature or at 37°C, or with or without agitation. Interestingly, TG1 grown previously in LB broth instead of LB agar and tested in the exponentially growing phase produced the most DNA fragmentation (class IV) after 0 min; i.e., immediately after the 8 min of microgel preparing.

**Figure 4:**
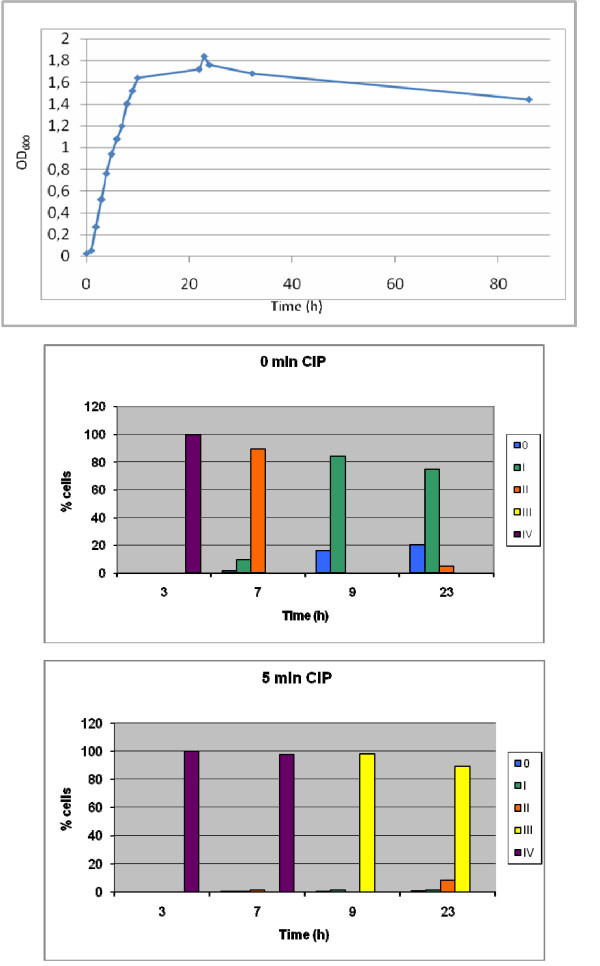
**DNA fragmentation in nucleoids from *E. coli *strain TG1 exposed to CIP in different culture times**. The growth curve of the bacteria, evaluated by monitoring turbidity at OD_600_, is presented above. The distribution of the frequencies of the diffusion widths of DNA fragments from the nucleoids were categorized into the five classes 0 to IV described in Table 1 and Fig. 2. Aliquots from a batch culture were removed at 3 h (exponentially growing phase) and at 7, 9, and 23 h (stationary phase), incubated with 1 μg/ml CIP for 0 (i.e., technical processing time of 8 min) **(medium) **and 5 min **(below)**, and then processed to determine the DNA fragmentation.

To investigate why the DNA damage level was dependent on the previous culture conditions, TG1 was grown in LB broth for 23 h, and the OD_600 _was monitored. Aliquots were removed after different culture times and incubated with 1 μg/ml CIP for 0 and 5 min (adding the 8 min of microgel preparation) (Fig. 4). After 3 h of culture (i.e., in the exponentially growing phase), all nucleoids were class IV after 0 and 5 min, as described above. After 7 h, the culture had achieved the stationary phase, and the nucleoids appeared mainly as class II (89.4%) and a few of them as class I after 0 min of incubation, whereas most (97.8%) were class IV after 5 min. Aliquots removed after 9 h (i.e., stationary phase) showed nucleoids as classes I (84.0%) and 0 (16.0%) after 0 min, and class III (98.4%) after 5 min incubation with CIP. The same result occurred after 23 h of culture. This experiment suggests that the growing conditions influence the speed of the CIP effect, which becomes increasingly slower when the bacteria are progressing into the stationary phase.

### Evolution of DNA damage

**Figure 5:**
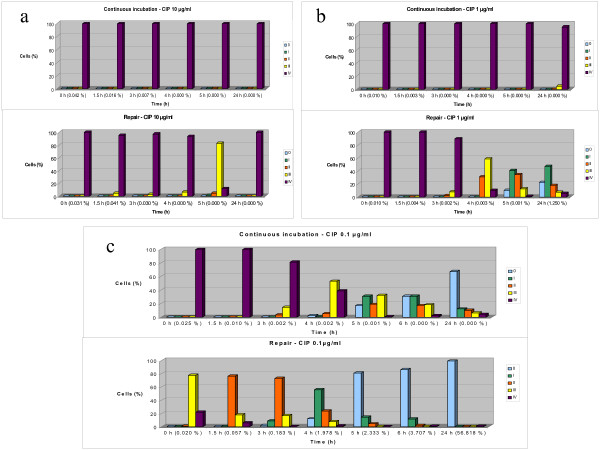
**Repair of CIP (10 μg/ml) induced DNA fragmentation**. The distribution of the frequencies of the diffusion widths from the nucleoids were categorized according to the five classes of fragmentation level (0 to IV) presented in Table 1 and Fig. 2. The different repair times after exposure of TG1 *E. coli *to three doses of CIP (**a: **10 μg/ml, **b: **1 μg/ml, and **c: **0.1 μg/ml) for 40 min are presented. Viability (%) is indicated next to each repair time. Each dose is shown with its respective culture (above) in which the antibiotic was present during the incubation time.

The TG1 *E. coli *strain was exposed to three different doses of CIP, 10, 1, and 0.1 μg/ml, for 40 min. After this treatment, the antibiotic was washed out, and the bacteria were incubated for 0, 1.5, 3, 4, 5, and 24 h (Fig. 5).

After exposure to the highest dose (10 μg/ml), all nucleoids were extremely fragmented, i.e., class IV. The DSB repair was limited and clearly noticeable only after 4 h; 82.5% of nucleoids were of class III after 5 h. Remarkably, all the nucleoids from the bacteria observed after 24 h showed massive fragmentation (class IV). Viability was very low after 0, 1.5, 3, and 4 h, and zero after 5 and 24 h (Fig. 5a).

Immediately after incubating with the 1 μg/ml dose, all nucleoids were class IV. A higher repair level was observed than after the highest dose, predominantly class III (58.7%) after 4 h, class I (41.0%) after 5 h, and class I (47.1%) after 24 h. Apparently repaired nucleoids without diffusing DNA fragments (10.2%) were visualized after 5 h, and this increased to 22.2% after 24 h. However, the viability was very low, as in the experiment with the highest dose (Fig. 5b).

In contrast to the results at the higher doses, repair activity was evident in the cultures exposed continuously to 0.1 μg/ml of CIP for the various times (Fig. 5c); 53.0% of nucleoids were class III after 4 h, and 31% were class I and 31% class 0 after 6 h. This latter time was assessed further in this experiment. The frequency of class 0 increased from 2.3% after 4 h to 67.3% after 24 h. In all cases, viability was very low or zero. Removing the drug resulted in faster repair kinetics, predominantly of class II (76.2%) after 1.5 h and class 0 (81.0%) after 5 h (Fig. 5c). The nucleoid pattern was similar to that of the untreated control cells after 24 h. Viability was initially very low, 2–4% after 4–6 h, and increased to 56.8% after 24 h (Fig. 5c). Thus, we found no clear relationship between the extent of repair of CIP-induced DNA breakage and cell viability.

### Evaluation of strains with known mechanisms of low sensitivity to CIP

**Figure 6:**
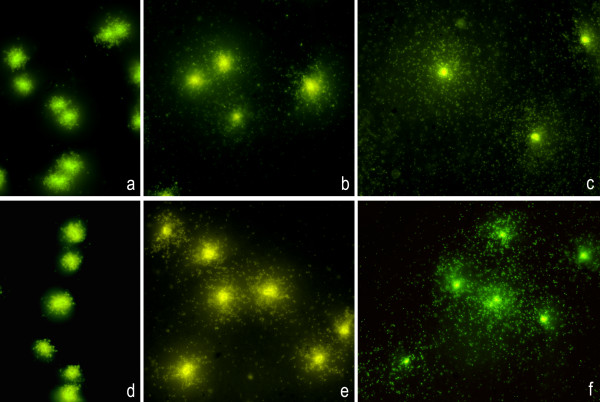
**Representative images of the DNA fragmentation induced by CIP in *E. coli *strains C-20 and C-15**. Left: MIC dose; medium: 10× MIC dose; right: 100× MIC dose. **Above: **control C-20 strain. **a: **0.007 μg/ml; **b: **0.07 μg/ml; **c: **0.7 μg/ml. **Below: **C-15 strain. **d: **0.25 μg/ml;**e: **2.5 μg/ml; **f: **25 μg/ml.

**Figure 7:**
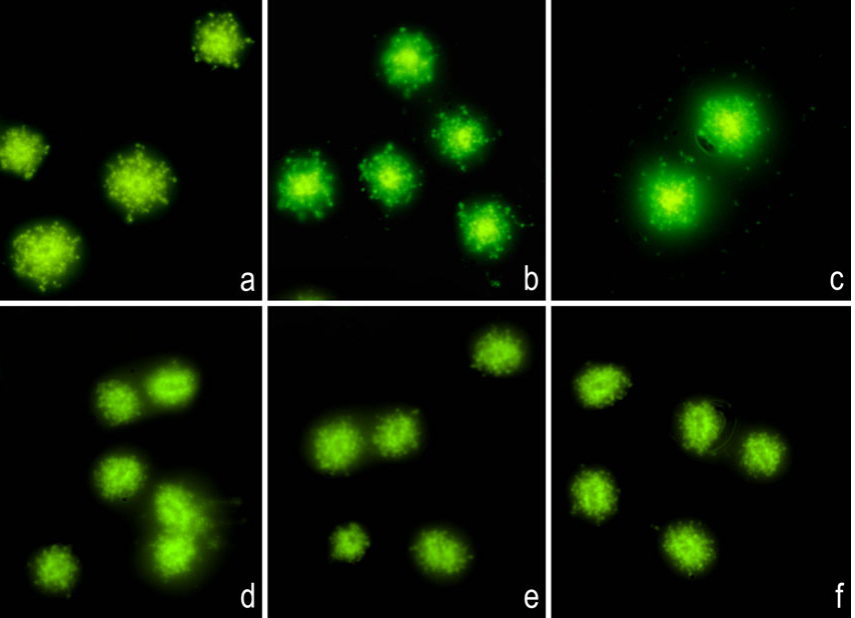
**Representative images of the DNA fragmentation induced by CIP in *E. coli *1273 and 1383 strains**. Left: MIC dose; medium: 10× MIC dose; right: 100× MIC dose. **Above: **1273 strain. **a: **8 μg/ml; **b: **80 μg/ml; **c: **800 μg/ml. **Below: **1383 strain. **d: **128 μg/ml; **e: **1280 μg/ml; **f: **12800 μg/ml.

The other *E. coli *strains used have been described previously [16]. They include strains with one amino acid substitution mutation in GyrA (C-15), two substitution mutations in GyrA (1273), and two substitution mutations in GyrA and another two in ParC (1383). The more mutations, the greater the resistance level, as reflected in the MIC values (Table 2). We also evaluated a strain with a qnrA1 plasmid (J53 qnrA1) [17] (Table 2). Doses lower than the MIC never resulted in visible DNA fragments. Thus, in strains with a MIC of 0.25 μg/ml (C-15 and J53 qnrA1), the MIC dose caused little DNA fragmentation in all bacteria (class I), whereas doses of 10× and 100× the MIC caused massive DNA fragmentation (class IV) (Table 2; Fig. 6). This behaviour is similar to that of the fully sensitive control strains but was shifted to a higher MIC. The 1273 strain did not show a clear effect at the MIC dose (8 μg/ml) but appeared as class I after 10× and class II after 100× of the MIC dose (Table 2; Fig. 7). The 1383 strain has a high MIC (128 μg/ml) and showed no DNA damage at any dose (Table 2; Fig. 7).

**Table 2:** DNA fragmentation levels obtained in strains of *E. coli *with different susceptibilities to CIP.

			CIP dose		
**Strain**	**Mutations**	**MIC**	**MIC 1×**	**MIC 10×**	**MIC 100×**

**C-20**	-	0.007	1.5 ± 0.3	6.7 ± 0.8	10.3 ± 2.5

**C-15**	Ser83Leu from GyrA	0.25	1.7 ± 0.3	6.2 ± 0.7	8.7 ± 1.1

**1273**	Ser83Leu and Asp87Tyr from GyrA	8	0	1.8 ± 0.3	2.7 ± 0.4

**1383**	Ser83Leu and Asp87Tyr from GyrA and Ser80Ile and Glu84Lys from ParC	128	0	0	0

**J53**	-	0.007	1.8 ± 0.8	9.2 ± 1.2	10.4 ± 2.0

**J53qnrA1**	Plasmid gene J53qnrA1	0.25	1.9 ± 0.4	9.5 ± 1.3	9.8 ± 0.9

The level of fragmentation obtained by different CIP doses is indicated by the width of the halo of dispersion of DNA fragments and is measured in μm (mean ± standard deviation). MIC is in μg/ml.

## Discussion

CIP-induced chromosomal DNA fragmentation was assayed in situ in *E. coli *using the Micro-Halomax^® ^kit [15]. We grew the samples in LB agar because this is simpler and is used routinely in clinical microbiology laboratories. The sample is scratched, diluted in LB broth to an OD_600 _of 0.05, and incubated with CIP in 4 ml of liquid LB in a 15 ml Falcon tube at 37°C with aeration. Incubation in a 1.5 ml Eppendorf tube with 24 μl of LB broth at room temperature (22°C) and without aeration does not modify the kinetics of DNA fragmentation induced by 1 μg/ml of CIP. We observed similar results in the TG1 strain and in three other *E. coli*-sensitive samples. Further confirmation in other sensitive strains could simplify the protocol for assessing *E. coli *sensitivity or resistance to CIP in the clinic.

Incubating TG1 with CIP for 40 min before technical processing produced a clear dose-response effect in chromosomal DNA fragmentation, and the damage level was similar in the different nucleoids. The effect on DNA was evident starting at the MIC dose, and DNA fragments were always visualized as spots of relatively small size, independently of the dose. The fragment size after oxolinic acid or norfloxacin treatment of *E. coli *has been estimated at 50 to 100 kb; i.e., the presumed size of the DNA loops of the nucleoid [18-20]. Our result suggests that the DNA fragments liberated from the nucleoid are of fairly regular size and that more fragments are released as the CIP dose increases. It also supports the possibility of clusters of preferential DNA gyrase cleavage sites [19]. It is possible that doses smaller than the MIC could induce a small amount of DSBs, which could be spaced widely in the different domains but not cause spreading of the fragments.

In our previous report, a CIP dose of 0.012 μg/ml produced slightly more damage than in the present study [15]. This is probably because of the harsher lysing conditions in our previous study, which may have caused additional DNA damage. This was corrected in the conditions used in our current study.

Adding 1 μg/ml of CIP to TG1 in LB broth and instantaneous processing using our technique produced just barely detectable DNA fragmentation. Taking TG1 from LB agar reduces the extent of damage. DNA damage increases progressively with incubation time, and a 30 min incubation is needed to achieve the maximum level of DNA fragmentation. Remarkably, when the bacteria came from exponentially growing cultures in LB broth, the highest DNA fragmentation level was observed immediately (0 min). These results suggest that the CIP effect on DNA is cumulative with time and that its velocity is dependent on the growing conditions. We confirmed this hypothesis by analyzing aliquots removed periodically from a batch culture incubated with 1 μg/ml CIP for 0 and 5 min. The DNA fragmentation level declined progressively as the bacteria proceed into the stationary phase.

Most *E. coli *cells divide uniformly in exponentially growing cultures but stop dividing when they achieve the stationary phase [21]. Bacteria grown in LB agar should be heterogeneous with regard to the growing phase, both exponential and stationary. The MIC is an average of the bacterial sensitivity to the antibiotic, which reflects the different effect of CIP on DNA. The DNA fragmentation yield is homogeneous among the nucleoids in exponentially growing TG1 but is slower and tends to be more heterogeneous in the stationary phase. This greater heterogeneity was evident after short incubation with 1 μg/ml CIP but tended to be homogeneous after 40 min of treatment.

Pulse field gel electrophoresis shows that the norfloxacin-induced fragmentation in *E. coli *nucleoids is low in the stationary phase of growth [20]. This phenomenon could reflect decreased drug uptake, increased drug efflux, downregulation of topoisomerases, or a more tightly packed nucleoid structure as demonstrated by atomic force microscopy [22]. Using our procedure, we have also observed more compacted nucleoids in the stationary phase. The most probable explanation is the activation of multidrug transporters that exclude fluoroquinolones, which is mediated by quorum-sensing signals. In fact, the quorum-sensing transcription factor SdiA from *E. coli *is regulated in a density-dependent manner, and its overproduction upregulates AcrAB, which increases resistance to quinolones and other antibiotics [23].

The lysing solution causes protein denaturation, so theoretically, the sensitivity-resistance assay is adequate to investigate sensitivity to fluoroquinolones at the relevant doses. CIP-mediated DSBs are natively unconstrained and are considered irreversible and lethal. In the case of first-generation quinolones such as nalidixic acid, the technique would artificially unconstrain DSBs that are naturally confined in the cleaved complex. If so, both reversible non-lethal DSBs and later lethal unconstrained DSBs should be detected without but cannot be differentiated in the assay. Addition of the chelating agent EDTA seems to reverse the cleaved complex formation by quinolones [7], possibly because incubation with EDTA before lysis allows the resealing of the reversible DNA breaks so that only the irreversible DSBs would be detected.

CIP-induced DSBs were not totally irreversible, and a progressive repair activity with time was evident in TG1. The magnitude of DNA repair was inversely related to dose and was noticeable after a dose of 0.1 μg/ml but scarce after a dose of 10 μg/ml. This repair was evident when the antibiotic was removed after the 40 min incubation and when TG1 was exposed continuously to the low dose (0.1 μg/ml) without CIP removal. The progressive spontaneous CIP degradation or inactivation with time in culture cannot be discounted, and the effect of CIP could be smaller despite being long lasting, especially if added at a low dose.

*E. coli *may repair DSBs by RecA-dependent homologous recombination (HR) [24]. CIP-induced DSBs could be processed to single-stranded DNA, a target for RecA, which promotes recombinatorial repair and induction of the SOS response through activation of the autocleavage of the LexA repressor [25,26]. Rapid lethality is increased by the *lexA *Ind-allele, and recombination-deficient *E. coli *strains are hypersensitive to quinolones [27]. The RecBCD nuclease/helicase also seems to be required for SOS induction by quinolones, as demonstrated with nalidixic acid [28]. Interestingly, DSBs may also be repaired by a non-homologous end joining (NHEJ) mechanism that comprises break recognition, end processing, and ligation activities. Although *E. coli *lacks a NHEJ pathway, its presence has been demonstrated in mycobacteria and bacillus [29]. Nevertheless, NHEJ deficiency caused by the loss of Ku and ligD has no effect on the sensitivity to quinolones of *Mycobacterium smegmatis *[30].

Repair of quinolone-induced DSBs probably needs more complex processing because both 5' ends of cleaved DNA are linked covalently via phosphotyrosine bonds to a topoisomerase subunit. These DNA-protein crosslinks (DPCs) could be eliminated in coordination with the nucleotide excision repair (NER) mechanism. The urvABC nuclease, which initiates the NER pathway in *E. coli*, may incise DPCs with or without previous proteolytic degradation of the crosslinked protein [31]. Accordingly, NER seems to be involved in CIP-induced DNA damage, as demonstrated in deficient *E. coli *strains [27]. Although both NER and HR may commit to the repair of DPCs, it has been proposed recently that DPCs with crosslinked proteins of sizes < 12–14 kDa are repaired by NER, whereas oversized DPCs are processed exclusively by RecBCD-dependent HR [32]. If confirmed, the later mechanism should be preferred in the repair of DPCs involving topoisomerase subunits.

The repair activity was not strictly related to viability. Although the nucleoid may appear normal after repair, particularly at the low dose (0.1 μg/ml), the bacteria may not be fully viable, possibly because of the lack of total fidelity in restitution and the SOS response, resulting in an error-prone repair [26]. Some misrepaired lesions could lead to a non-viable cell. The DNA repair experiments emphasize the importance of achieving the necessary concentrations over a prolonged time for the successful clinical effect of quinolones.

DNA repair is not cited as a mechanism of decreased sensitivity to quinolones. Nevertheless, *E. coli *mutants with constitutive RecA expression or defective SOS induction may survive longer [27]. It is possible that dysfunction of certain DNA repair processes may lead to a low sensitivity to CIP, and this could increase the effect of other coexisting mechanisms of resistance. This possibility needs to be explored.

It is expected that resistance to fluoroquinolones would hinder the production of DSBs, which are slowly or rarely produced. Because DSBs appear to correlate strongly with the MIC and viability, the DNA fragmentation assay should detect resistance accurately. The preliminary study of the DNA fragmentation analysis in the four *E. coli *strains with low sensitivity to CIP suggests that this is the case. The 1273 strain did not show a clear effect at the MIC dose and had a lower DNA fragmentation level than that observed in other strains at the same multiple of MIC dose. This phenomenon could be related to the accumulation of multiple resistance mechanisms, such as multiple mutations in different topoisomerase subunits and in conjunction with altered outer membrane proteins and lipopolysaccharide, and increased activity of efflux systems [33]. Since only J-53 and J-53qnrA1 strains are isogenic, the other strains could have other differences that could influence the results. Moreover, the growth inhibition may not be dependent on inhibition of the topoisomerases leading to DNA fragmentation and the possibility exists of unknown mechanisms of action.

## Conclusion

The DNA fragmentation assay may be a simple and rapid test to evaluate the sensitivity and resistance to quinolones. We are currently performing more comprehensive assessment of different characterized CIP-resistant and CIP-sensitive *E. coli *strains and in clinical samples.

## Authors' contributions

MT and RB performed technical experiments and statistical analysis. JG participated in image acquisition and image analysis. GB participated in the design of the study and data analysis. JLF conceived the study, participated in its design and coordination and wrote the initial draft of the manuscript. All authors read and approved the final manuscript.
